# Primary sclerosing cholangitis

**DOI:** 10.1186/1750-1172-1-41

**Published:** 2006-10-24

**Authors:** Joy Worthington, Roger Chapman

**Affiliations:** 1 Department of Gastroenterology, John Radcliffe Hospital, Headington, Oxford, OX3 9DU, UK

## Abstract

Primary sclerosing cholangitis (PSC) is a chronic cholestatic liver disease of unknown aetiology characterised by inflammation and fibrosis of the biliary tree. The mean age at diagnosis is 40 years and men are affected twice as often as women. There is a reported annual incidence of PSC of 0.9–1.31/100,000 and point prevalence of 8.5–13.6/100,000. The onset of PSC is usually insidious and many patients are asymptomatic at diagnosis or have mild symptoms only such as fatigue, abdominal discomfort and pruritus In late stages, splenomegaly and jaundice may be a feature. In most, the disease progresses to cirrhosis and liver failure. Cholangiocarcinoma develops in 8–30% of patients. PSC is thought to be immune mediated and is often associated with inflammatory bowel disease, especially ulcerative colitis. The disease is diagnosed on typical cholangiographic and histological findings and after exclusion of secondary sclerosing cholangitis. Median survival has been estimated to be 12 years from diagnosis in symptomatic patients. Patients who are asymptomatic at diagnosis, the majority of whom will develop progressive disease, have a survival rate greater than 70% at 16 years after diagnosis. Liver transplantation remains the only effective therapeutic option for patients with end-stage liver disease from PSC, although high dose ursodeoxycholic acid may have a beneficial effect.

## Disease name

Primary sclerosing cholangitis (PSC).

## Definition

Primary sclerosing cholangitis (PSC) is a chronic cholestatic liver disease of intra and/or extrahepatic bile ducts. A concentric obliterative fibrosis occurs which leads to bile duct strictures, which in turn can progress to biliary cirrhosis and liver failure. Cholangiocarcinoma develops in 8–30% of patients [1,2].

Secondary sclerosing cholangitis (SSC) is a sclerosing cholangitis that occurs secondary to a known pathogenic process or injury such as obstruction of the bile ducts due to tumour or gallstones; surgical, chemical or ischaemic injury; bacterial infections or congenital abnormalities. SSC has clinical features similar to those of PSC.

## Associated diseases

### Primary association

Inflammatory bowel disease.

### Others

Coeliac disease, diabetes mellitus, rheumatoid arthritis, Sjogren's disease, systemic sclerosis, chronic pancreatitis, cystic fibrosis, retroperitoneal fibrosis, sarcoidosis, thyroiditis, systemic lupus erythematosus (SLE), lupus nephritis, autoimmune haemolytic anaemia, idiopathic thrombocytopenic purpura, Langerhans cell histiocytosis, membranous nephropathy, Peyronie's disease, vasculitis, gallbladder disease.

### Complications

Colorectal cancer, cholangiocarcinoma.

## Epidemiology

PSC occurs with a 2:1 male predominance. Little is known about the incidence and prevalence of PSC. There is a reported annual incidence of PSC of 0.9–1.31/100,000 and point prevalence of 8.5–13.6/100,000, which appears to be increasing [3-5]. This probably reflects ascertainment bias rather than a true increase. Patients usually present in the third to fifth decade but PSC is recognised as a cause of chronic liver disease in children. It is also unusual to find more than one family member affected.

PSC is frequently associated with inflammatory bowel disease (IBD), especially ulcerative colitis and, less often, with colonic Crohn's disease. Approximately three quarters of Caucasian patients with PSC have IBD [6,7] and 2–7.5% of patients with ulcerative colitis have PSC [8-10]. This may be an underestimate as patients with PSC may have normal liver function tests. The symptoms of IBD may predate or follow the diagnosis of PSC. The clinical course of ulcerative colitis in PSC patients is often mild and there is a higher prevalence of rectal sparing compared with controls (52% *vs *6%). There is also a higher prevalence of backwash ileitis (51% *vs *7%) [11]. The risk of colonic dysplasia and colorectal cancer is higher in those with PSC. A long history of IBD is also a risk factor for cholangiocarcinoma development. Colectomy does not alter the course of PSC [12].

## Clinical description and diagnostic methods

The diagnosis of PSC is based on a combination of clinical features and cholestatic liver function tests with typical cholangiographic findings and confirmed by characteristic histological abnormalities. In small duct disease, patients have typical biochemical and histological features but a normal cholangiogram. All secondary causes of cholangitis and other causes of liver disease should be excluded.

### Clinical features

The clinical course of PSC is highly variable. Patients may present with abnormal liver function tests on a background of IBD. Patients may remain asymptomatic for years or may develop symptoms of fatigue, abdominal discomfort, pruritus and weight loss. Ultimately patients may develop liver failure or cholangiocarcinoma.

Examination may be normal early in disease. As the disease progresses, patients may develop hepatomegaly. In late stages splenomegaly and jaundice may be a feature. Signs of liver failure and portal hypertension are late signs.

### Laboratory tests

Alkaline phosphatase is usually raised and transaminases and gamma-glutamyl transpeptidase (GGT) can also be elevated. Patients with PSC often have marked fluctuations in liver function tests and these are not predictive of prognosis [29]. Results of hepatic synthetic function, *i.e*. serum albumin and prothrombin time (PT), become abnormal with advanced disease.

Some immunological tests may help in the diagnosis of PSC. Hypergammaglobulinaemia occurs in a third of patients and immunoglobulin M (IgM) levels are increased in 50% of patients with advanced disease.

### Imaging

Once abnormal biochemistry is revealed on the liver function tests (LFTs), diagnosis is made mainly on the basis of the cholangiogram. A magnetic resonance cholangiogram (MRCP) is being used more frequently for diagnosis and endoscopic retrograde cholangiography (ERCP) is performed more as a therapeutic procedure.

**Figure 1:**
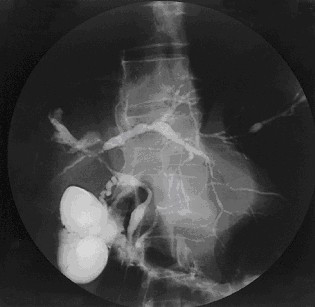
Cholangiogram of primary sclerosing cholangitis.

Typical cholangiographic findings show diffuse stricturing of intrahepatic and extrahepatic bile ducts with dilatation of the areas in between (See Figure 1).

More advanced disease is associated with long strictures. The presence of a dominant stricture should raise the possibility of a cholangiocarcinoma as a complication of PSC. In small duct disease the cholangiogram will be normal. Ultrasound can be helpful in excluding other causes of biliary obstruction such as gallstones and malignancy.

### Histology

**Figure 2:**
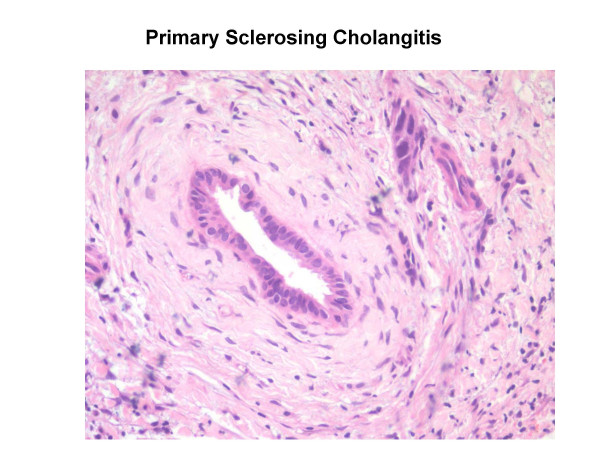
Histological features of primary sclerosing cholangitis.

In early stages, changes associated with PSC can be focal and may be missed on liver biopsy or the changes may be non-specific. The characteristic finding is of concentric "onion-skin" fibrosis surrounding the bile ducts (See Figure 2). Other bile duct abnormalities may include necrosis of epithelial cells, inflammatory infiltrates and fibrosis. There may be intrahepatic bile duct proliferation with ductopaenia or oedema in some portal tracts. In advanced cases, loss of bile ducts can be a feature (vanishing bile duct syndrome). The parenchyma usually shows non-specific changes [30]. Copper storage protein may be seen in advanced cases.

## Differential diagnosis

Differential diagnosis should exclude other liver diseases such as autoimmune hepatitis (can coexist with PSC, termed overlap syndrome), primary biliary cirrhosis, viral hepatitis, as well as secondary causes of cholangitis.

## Aetiology and pathogenesis

The aetiology and pathogenesis of PSC are still unknown. The following may be important in the development of PSC.

### Immunology

Lymphocytic portal tract infiltration and associations with human leukocyte antigen (HLA) haplotypes and autoantibodies are suggestive of an immune mediated basis for this disease. Autoimmune diseases are more frequent in patients with PSC than in patients with IBD who do not have PSC. The most common are diabetes mellitus, coeliac disease and Grave's disease [17]. Rheumatoid arthritis has also been described in association with PSC and may be a clinical marker for those at high risk of rapid progression to cirrhosis [18].

A high prevalence of atypical anti-neutrophil cytoplasmic antibodies (ANCA) are present in the serum in PSC (33–88%). They are also found in ulcerative colitis (60–87%) and type I autoimmune hepatitis (50–96%) [17,18]. These antibodies are not involved in the pathogenesis of PSC and probably represent an epiphenomenon. Other autoantibodies can be detected in patients with PSC but their presence is not useful for diagnosis or prognosis and, therefore, is of unclear relevance.

Hypergammaglobulinaemia occurs in one third of patients with PSC and IgM levels are increased in 50% of advanced cases [19]. Other immunological changes include a decrease in circulating T cells and an increased ratio of CD4 to CD8 cells.

Grant *et al*. hypothesised that T Lymphocytes derived from the inflamed gut act as long-lived memory cells [20]. They enter the enterohepatic circulation to gain access to the liver and cause disease when then activated by a certain stimulus [20].

### Genetics

PSC is probably acquired through inheriting a combination of genetic polymorphisms that interrelate to cause disease susceptibility. Six different HLA molecules have so far been associated with PSC [21]. Non-MHC (major histocompatibility complex) genes may also play a part but data remains controversial. The importance of genetics in PSC is not fully understood and still being actively researched.

### Infections/toxins

The association of PSC and IBD led to Vierling's hypothesis that PSC may be triggered by infections or toxins acting as molecular mimics entering the portal circulation through a permeable colon [22]. The Kupffer cells are activated and cause activated neutrophils, macrophages, lymphocytes and fibroblasts to be attracted, which in turn could then lead to ischaemia, atrophy, cholestasis, fibrosis and then cirrhosis of the biliary cells. However, as PSC can run an independent course from IBD, this suggests bacteraemia on its own is not the cause of PSC development.

### Smoking/surgery

There is a well established increased risk of ulcerative colitis in non-smokers when compared to controls. PSC also seems to be a disease of non-smokers, independently of whether the patient has IBD. Previous tonsillectomy may be associated with a decreased risk of PSC. Whilst appendicectomy is not associated with a decreased risk, it may be associated with a delayed onset of PSC. Further studies are required to analyse this association [23-26].

## Management including treatment

Since the aetiology and pathogenesis of PSC are still unknown, establishing treatment strategies is difficult. Treatments should ultimately improve symptoms and aim to improve survival. Liver transplant remains the only definitive treatment.

### Ursodeoxycholic acid

Ursodeoxycholic acid (UDCA) is a naturally occurring bile acid that improves liver enzyme function in PSC but its effect on liver histology remains controversial. In PSC, there is evidence that high doses (20–30 mg/kg/d) of UDCA may be more effective than moderate doses (10–15 mg/kg/d) at slowing progression of liver fibrosis and cholangiographic appearances [27,28]. The results of a controlled trial with high dose UDCA for the treatment of PSC has shown a trend towards a prolonged survival in the high dose UDCA group [29]. Studies using immunosuppressant therapy in combination with UDCA show some beneficial effects and need further analysis, as does treatment with antibiotics. In a few patients, features of both PSC and autoimmune hepatitis (AIH) may be present and this is one of the so-called overlap syndromes. This overlap syndrome, in particular, may be more common in children. Immunosuppression appears to be beneficial in this group [30-32].

#### Dysplasia and cancer

The risk of colorectal dysplasia and colorectal cancer is increased in those with ulcerative colitis who have PSC [33]. Although not evidence-based, current consensus is that these patients should be entered into a surveillance programme and undergo yearly colonoscopies once the diagnosis of PSC has been made. UDCA treatment in patients with PSC and ulcerative colitis has been shown to decrease the risk of colorectal dysplasia and colorectal cancer [34,35].

Cholangiocarcinoma develops in 8–30% of adult patients with PSC. Hepatocellular carcinoma and gallbladder carcinoma have also been described in PSC. There is also a suggestion of an increased risk of pancreatic carcinoma. Hepatobiliary malignancy is only detected in a proportion of those it is suspected in [36]. Of those PSC patients with cancer, one third will already have hepatobiliary malignancy at the time of or within a year after PSC diagnosis. The risk of hepatobiliary carcinoma is constant after the first year of PSC diagnosis with an incidence rate of 1.5% per year [37]. Measurement and detection of elevated CA19-9 may be a signal to investigate further, but it can also be raised in benign biliary disease [23,38]. Biliary Mac-2 binding protein has been suggested as a novel diagnostic marker for biliary tract carcinoma, especially when used in combination with biliary CA19-9 levels [39]. Early detection of cholangiocarcinoma is difficult as it may be indistinguishable on cholangiogram from a benign dominant stricture. Brush cytology of dominant strictures has not been shown to have a predictive value [40].

#### Other complications

Pruritus is a frequent complication and can be disabling. Agents such as cholestyramine (4–16 g/d), rifampicin or naltrexone may all be used [41,42]. Extra corporeal albumin dialysis has been tried in small numbers of patients with cholestasis, and appears to be an effective alternative for the treatment of patients with pruritus who do not respond to other therapeutic methods [43].

People with advanced PSC are often deficient in the fat-soluble vitamins A, D, E, K and replacement therapy can be given. In osteoporosis, physical exercise and maintenance of adequate calcium and vitamin D may be sufficient in the early stages. Patients with more advanced disease, especially those with IBD, should undergo bone density scanning. Bisphosphonates may be of value.

Recurrent bacterial cholangitis is common, especially in those with strictures, and should be treated with broad-spectrum antibiotics such as ciprofloxacin, which has a high biliary tract penetration. Prophylaxis with oral quinolones may decrease the frequency of cholangitis in those who have a history of recurrent episodes. Cholelithiasis and choledocholithiasis probably occur secondary to chronic cholestasis. ERCP can be used for balloon dilatation or stent placement in the treatment of dominant strictures, and may provide relief of symptoms [44].

### Surgery

Liver transplant is an option in the treatment of PSC for those who develop advanced disease. It is difficult to evaluate these patients with regard to timing of transplantation, as disease course is unpredictable. At least 20% of patients undergoing liver transplantation develop recurrent PSC within 5 years [45]. The 5-year survival post transplant is around 75% [46]. The presence of cholangiocarcinoma is traditionally a contraindication to transplant but one study shows a 5 year survival of around 35% for those with cholangiocarcinoma. Thus, if cholangiocarcinoma is only suspected and not definitely diagnosed, the patient should be considered for transplant. Incidental finding of a cholangiocarcinoma during transplant does not affect patient survival significantly [36,47]. Inflammatory bowel disease symptoms may become more severe post transplantation prompting proctocolectomy, and 5–10% of those with IBD develop colorectal cancer after liver transplant [48].

## Prognosis

Median survival has been estimated to be 12 years from diagnosis in symptomatic patients [1,6,7]. Patients who are asymptomatic at diagnosis, the majority of whom will develop progressive disease, have a survival rate greater than 70% at 16 years after diagnosis [49].

Patients who have cholestatic liver function tests and a clinical diagnosis of PSC with features consistent with this diagnosis on the liver biopsy, but who have a normal cholangiogram, have small duct PSC; 6–11% of the PSC population have small duct disease. Patients with small duct disease follow a benign course and have a favourable prognosis with regard to survival, transplantation and development of cholangiocarcinoma. The small duct disease does, however, progress in a small number (12%) to large duct disease [50-52].

## Unresolved questions

The aetiology of PSC is still unknown.

More needs to be known about the natural history of PSC.

The availability of predictors for development of colonic and bile duct cancer would be useful.
